# Comparative the efficacy and acceptability of immunosuppressive agents for myasthenia gravis: A protocol for systematic review and network meta-analysis

**DOI:** 10.1097/MD.0000000000031454

**Published:** 2022-12-16

**Authors:** Peng Xu, Ying Zhang, Tianying Chang, Li Jiang, Zhiguo Lv, Yibin Zhang, Hanying Xu, Dongmei Zhang, Tianye Lan, Yingzi Cui, Zhen Hua, Chengfei Gao, Jing Lu, Qingxia Huang, Jinhui Tian, Jihui Ma, Jian Wang

**Affiliations:** a Department of Neurology, The Affiliated Hospital of Changchun University of Chinese Medicine, Changchun, Jilin Province, China; b GCP Department, The Affiliated Hospital to Changchun University of Chinese Medicine, Changchun, Jilin Province, China; c College of Traditional Chinese Medicine, Changchun University of Chinese Medicine, Changchun, Jilin Province, China; d Scientific Research Office, The Affiliated Hospital to Changchun University of Chinese Medicine, Changchun, Jilin Province, China; e Department of Cardiology, The Affiliated Hospital of Shandong University of Traditional Chinese Medicine, Jinan, Shandong Province, China; f Department of Physical Medicine and Rehabilitation, The Affiliated Hospital of Qingdao University, Qingdao, Shandong Province, China; g Department of Biomedical Engineering, The Hong Kong Polytechnic University, Hong Kong, China; h Research Center of Traditional Chinese Medicine, The Affiliated Hospital to Changchun University of Chinese Medicine, Changchun, Jilin Province, China; i Evidence-Based Medicine Center, Lanzhou University, Lanzhou, Gansu Province, China; j Department of Health Research, Evidence, and Impact, McMaster University, Hamilton, ON, Canada.

**Keywords:** Bayesian network meta-analysis, immunosuppressive drugs, immunotherapy, myasthenia gravis, protocol, systematic review

## Abstract

Immunosuppressive drugs are routinely used to treat myasthenia gravis (MG). However, current recommendations provide limited evidence to support treatment options, leading to considerable variation in practice among healthcare specialists. Hence, we present a protocol for a systematic review and network meta-analysis (NMA) to update the evidence by comparing the efficacy and acceptability of oral immunosuppressive drugs for the treatment of MG.

**Methods:**

We will conduct a systematic review and NMA of all randomized controlled trials evaluating the following oral immunosuppressive drugs for the treatment of MG. Published studies will be searched using the following databases from inception to November 23, 2021: CENTRAL, the CINAHL, MEDLINE, Embase, PsycINFO, Web of Science, and 3 Chinese databases (Chinese Biomedical Literatures Database, CNKI, and Wan Fang database). Assessment of study eligibility and data extraction will be conducted independently by 2 reviewers. The main outcome will be a quantitative MG scoring system. We will conduct Bayesian NMA to synthesize all evidence for each outcome and obtain a comprehensive ranking of all treatments. The quality of the evidence will be evaluated using the Grading of Recommendations, Assessment, Development, and Evaluations framework.

**Results:**

The objective of this study was to assess the relative clinical efficacy and acceptability of first-line immunosuppressants for the treatment of MG, using a systematic review and NMA approach.

**Conclusion:**

In the absence of head-to-head trials comparing therapies, evidence from this NMA of available clinical trials will inform clinicians, patients, and families the risk-benefit profiles of different treatment options.

## 1. Introduction

Myasthenia gravis (MG) is a prototypical antibody-mediated autoimmune disease characterized by fluctuating muscle weakness and fatigue on exertion that results from autoantibodies to proteins of the neuromuscular junction (NMJ).^[1]^The incidence of MG in the total population is rare, it often causes chronic and, severe disability, and has a high mortality rate.^[2]^ It poses a substantial challenge for health systems in both developed and developing countries, with the need to treat patients, optimize resources, and improve overall health care for rare diseases.

The current standard treatment for MG includes symptomatic treatment (acetylcholinesterase inhibitors), thymectomy, first-line immunomodulation (plasma exchange and, subcutaneous or intravenous immunoglobulins), and immunosuppressive drugs.^[3]^ Most patients with MG require prolonged and even life-long immunosuppressive medication to achieve the treatment goals of full or nearly complete physical function, as symptomatic drug monotherapy is usually insufficient to achieve disease control.^[4]^ Grouped into various classes of drugs with slightly different mechanisms of action, most guidelines recommend that long-acting immunosuppressive agents for MG include azathioprine (AZA), mycophenolate mofetil (MMF), cyclosporine, tacrolimus (FK-506), methotrexate, and cyclophosphamide.^[3,5–7]^ Biological therapy is now common in many centers. Complement modulation treatment and other emerging therapies have been used for refractory MG. Although recommendations in the management of MG are still not updated, they remain to be discussed. No consensus has been reached on the ideal therapeutic algorithm for MG.^[8,9]^

As the arsenal of MG immunosuppressive therapies increases, choosing a treatment that is targeted at the individual patient becomes more difficult. Only a few immunosuppressive drugs have been tested in larger randomized controlled trials providing unequivocal class I evidence for their use in patients with MG.^[10]^ Furthermore, some recent randomized, controlled trials have shown significant heterogeneity in treatment response.^[11–13]^

To make informed prescribing decisions, clinicians need to understand the benefits and risks of immunosuppressive treatments. Several traditional meta-analyses have evaluated the efficacy and safety of single or double intervention.^[14–16]^ However, to the best of our knowledge, the comparative effectiveness, unacceptability of treatment, and safety profiles of all available immunosuppressive drugs for MG have not been elucidated. There is a major unmet understanding of the relative efficacy of different immunosuppressive agents in the treatment of MG. Network meta-analyses (NMA) of existing datasets enable the synthesis of direct and indirect evidence across a network of randomized trials to infer the relative effectiveness of multiple interventions.^[17]^ Therefore, the NMA will be performed to compare the efficacy and acceptability of the major immunosuppressants administered, that is, AZA, MMF, cyclosporine, FK-506, methotrexate, and cyclophosphamide, in the treatment of MG. In the absence of head-to-head trials directly comparing therapies, evidence from this NMA will inform clinicians as well as patients and families as they consider the risk-benefit profiles of different treatment options.

## 2. Materials and methods

This protocol adhered to the Preferred Reporting Items for Systematic Review and Meta-Analysis Protocols statement (PRISMA-P).^[18]^ This protocol has been registered in the International Prospective Register of Systematic Reviews (PROSPERO) (the registration number is CRD42018117022). All subsequent amendments to the protocol have been clarified in the final manuscript.

The final results of the NMA will be reported according to the PRISMA 2020 statement^[19]^; and the PRISMA extension for network meta-analyses (PRISMA-NMA).^[20]^

The data that will be used in this NMA are neither individual nor private. Therefore, NMA does not require ethical approval or informed consent. The results of this study will be published in a peer-reviewed journal.

### 2.1. Inclusion criteria for studies

The eligibility criteria will meet the PICOS (participant, intervention, comparator, outcomes, and study design) structure.

#### 2.1.1. Types of participants (P).

We included participants diagnosed with MG according to standard operationalized diagnostic criteria.^[3,5,6]^ There will be no restrictions on the sex, age, or race of the patient. Patients with allergies, serious complications (cardiovascular diseases, renal insufficiency, or other severe systemic diseases), and Lambert–Eaton myasthenia syndrome were also excluded from this review. Pregnant or lactating women were excluded from the study.

#### 2.1.2. Types of interventions (I).

We focused on comparing the first-line oral immunosuppressive agents recommended by international and national treatment guidelines^[3,5,6]^: AZA, MMF, methotrexate, cyclophosphamide, cyclosporine, and FK-506.

#### 2.1.3. Types of comparators (C).

The control interventions included placebo or any other active immunosuppressive drug regardless of the route of delivery (such as oral or enema), dosage, frequency, and duration.

We will include 3 types of control interventions:

(1) One immunosuppressive drug alone versus placebo.
(2) One immunosuppressive drug alone versus another active immunosuppressive drug.
(3) Combination of 2 immunosuppressive drugs versus active treatment alone.

#### 2.1.4. Outcome measures (O).

##### 2.1.4.1. Primary outcomes.

The primary outcome will be the change from baseline in the quantitative MG scoring system (QMGs), which is essential in the objective evaluation of MG therapy.^[21]^ The QMG score is a 13-item categorical scale that assesses muscle weakness, with each item scored from 0 to 3 points. A total score of 0 represents no weakness, and a score of 39 represents severe weakness. Improvements in the QMG score of 2 to 3 points may be considered clinically meaningful depending on the baseline disease severity.^[22]^

##### 2.1.4.2. Secondary outcomes.

(1) The key secondary end point was the change from baseline in the MG activities of daily living (MG-ADL) score. The MG-ADL is an 8-item categorical scale that assesses the effect of MG on daily functions that are typically affected by the disease, with each item scored from 0 to 3 points. A total score of 0 represents normal function, and a score of 24 represents a severe effect on activities of daily living from MG (total score of 0–24).
(2) The 2 safety outcomes included withdrawal from treatment due to adverse events (acceptability) and the occurrence of serious adverse events. For these outcomes, we will rely on reporting these terms in the trial publications. Where adverse event rates in those specific categories are not given in the report, we will contact the authors for the data. For each safety outcome, we will extract the sample size for each treatment and number of patients experiencing the event.

#### 2.1.5. Types of studies (S).

This systematic review will include randomized controlled trials comparing immunosuppressive agents with placebo or other active immunosuppressive agents as an oral monotherapies for MG. No restrictions will be imposed on language.

### 2.2. Exclusion criteria

Non-randomized studies will be excluded from this review.

### 2.3. Search methods for identification of studies

We will conduct a systematic search of the following electronic databases from inception to November 2021: Medline (by PubMed), EMBASE, the Cumulative Index to Nursing and Allied Health Literature (CINAHL), the Cochrane Central Register of Controlled Trials (CENTRAL), Web of Science, Chinese Biomedical Literature Database, and China National Knowledge Infrastructure (CNKI). The search strategy is presented in Table 1.

**Table 1:** Search strategy in PubMed.

Number	Search items
#1	“Myasthenia Gravis”[Mesh] OR (Myasthenia Gravis, Ocular) OR. (Ocular Myasthenia Gravis) OR (Myasthenia Gravis, Generalized) OR (Generalized Myasthenia Gravis) OR (Early-onset Myasthenia Gravis) OR (Late-onset Myasthenia Gravis)
#2	“ azathioprine” [ti, ab] OR “ mycophenolate mofetil “ [ti, ab] OR “ methotrexate “ [ti, ab] OR “ cyclophosphamide “ [ti, ab] OR “ tacrolimus “ [ti, ab]
#3*	randomized controlled trial [pt] OR controlled clinical trial[pt] OR randomized [tiab] OR. placebo [tiab] OR clinical trials as topic [mesh:noexp] OR randomly[tiab] OR trial [ti]
#4	#1 AND #2 AND #3
#5	NOT (“animals”[mesh] NOT “humans”[mesh])
#6	#4 AND #5

* Direct link to PubMed with sensitivity- and precision-maximizing version (2008 revision). Lefebvre C, Manheimer E, Glanville J. Chapter 6: Searching for studies. In: Higgins J, Green S (eds). Cochrane Handbook for Systematic Reviews of Interventions. Version 5.1.0 (updated March 2011). The Cochrane Collaboration, 2011. Available from: www.cochrane-handbook.org.

We will search the following sources to identify clinical trials, either in progress or completed: reference lists of identified articles for inclusion, Google Scholar, Baidu Scholar, Clinical Trials.gov, and the WHO International Clinical Trials Registry Platform.

### 2.4. Selection of studies

**Figure 1.**
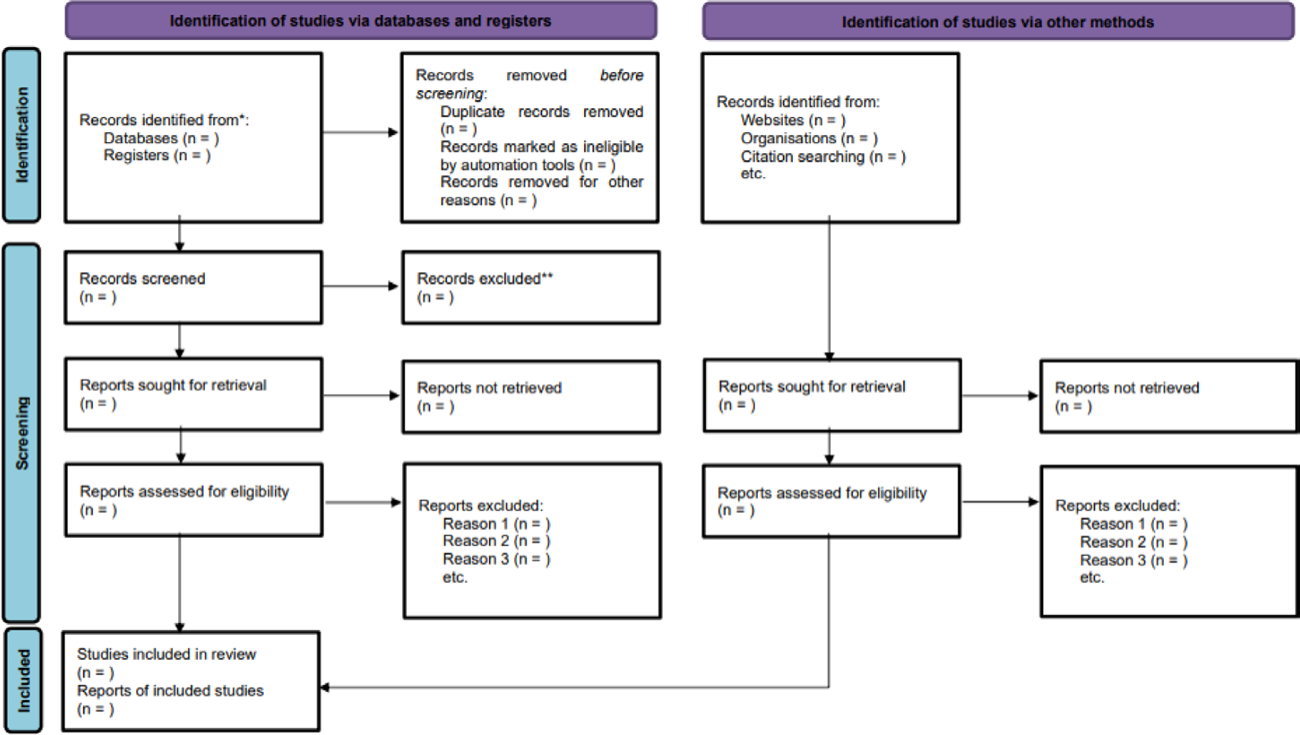
Flow diagram of study selection process.

The search results will be imported into the EndNote software (V.X9, Thomson Reuters). After initial screening of the title and abstract, we will screen the full text of all potentially eligible trials. Four independent reviewers (TL, LJ, QH, and YZ) will sequentially eligible studies by screening the titles, abstracts, and full texts. These 4 reviewers will act as pairs of reviewers. The reasons for trial exclusion will be documented in detail during full-text screening, and any disagreements will be resolved at the third reviewer (DZ). If necessary, methodological experts (JT) will be consulted to reach a consensus. The process of selecting the studies will be shown using the PRISMA-compliant flow chart (Fig 1).

### 2.5. Data extraction

Six reviewers (YZ, ZH, CG, TC, HX, and JL) will independently extract data from each included study and enter them in electronic forms in Microsoft Access 2010. The data extracted from each study include the bibliographic details (including the author’s name, publication year), patient characteristics (such as age, sex, and MG type), details of interventions and comparators (the route of administration, dosage, and frequency of the drug therapies and duration of treatment), and outcomes (Table 2). We will try to contact the corresponding authors for missing data or clarification of unclear information.

**Table 2:** Summary of the included randomized controlled trials.

**Item**	**Content**
**Publication details**	First-listed author, year of publication, country of conduct, funding sources (for profit, mixed, and nonprofit)
**Study characteristics**	Study design (randomized or nonrandomized)
	Setting (single or multicenter)
	Length of follow-up, accrual period
	Inclusion and exclusion criteria
	Rates of loss to follow-up (with reasons) number of study arms, number of patients randomized per arm
	Number of patients analyzed per arm
**Participant characteristics**	Age, the sample size of each arm, time since onset
	Number of patients with thymoma per arm. Number of patients with thymectomy per arm. Number of patients with acetyl-choline receptor antibodies per arm
**Intervention characteristics**	Dose, frequency, duration, the timing for the start of treatment, and adherence
**Outcomes of interest**	The reduction of quantitative MG scoring systems
	The reduction of glucocorticoids
	Adverse events
	The MG activities of daily living

### 2.6. Risk of bias assessment

For each eligible trial, after training and calibration exercises, 2 reviewers (PX and ZL) will independently assess the risk of bias in the included studies using the Cochrane Collaboration Risk of Bias in Randomized Trials (RoB 2.0 tool).^[23]^ We will evaluate the risk of bias in the following domains: bias from randomization, bias from deviations from intended interventions, bias due to missing data, bias in outcome measurement, and bias in the selection of the reported result. The sources of bias in each trial will be assessed and classified as “high risk,” “low risk,” or “unclear risk.” Any discrepancy in the assessment of the risk of bias will be resolved by discussion or third-party (YC, JM, and WJ) adjudication if needed.

### 2.7. Data synthesis and analysis

An overview of all selected studies will be narratively displayed. Once the data are obtained, a sheet will be made to tabulate the data for the different outcomes. Classification according to the population and study characteristics and nature of the therapy will be performed. Both traditional pairwise and network meta-analyses will be conducted.

### 2.8. Pairwise meta-analyses

Initially, we will perform a pairwise meta-analysis of interventions assessed head-to-head included in this analysis. For each pair-wise comparison, we will synthesize data to obtain summary standardized mean differences (Cohen *d*) for continuous outcomes or summary odds ratios for dichotomous outcomes, both with 95% credible intervals. For outcome measures investigated by 2 or more studies, if there is no clinical heterogeneity (e.g., different age or intervention) or methodological heterogeneity (e.g., different measurement tools), statistical heterogeneity will be assessed using the chi-square test for heterogeneity and quantified using the *I*^2^ statistic. If the *P* value is ≥ .1 and I2 ≤ 50%, we will synthesize standardized mean differences or OR using the fixed-effects model. If the *P*-value is < .1 and *I*^2^ > 50%, the random-effects model will be used.

### 2.9. Assessment of transitivity

Transitivity is the fundamental assumption of the NMA, which allows for valid indirect inference. All indirect analyses are based on the underlying assumption that the study populations in the trials being compared are sufficiently similar to be pooled, akin to meta-analyses.^[24]^ If the network is substantially intransitive, a joint analysis of treatments can be misleading. However, as it is difficult to identify transitivity using statistical analysis, the assessment will be based on clinical and methodological characteristics including participant characteristics (such as age, sex, and MG severity at baseline), study designs (blind method and risk of bias) and interventions (dosing schedule). All of these research aspects and influential factors will be investigated and reported.

### 2.10. The network meta-analysis

In the absence of important intransitivity, we will then perform network meta-analysis (NMA) that combining direct and indirect comparisons in a Bayesian hierarchical model.

We will generate posterior samples using Markov Chain Monte Carlo algorithm by applying JAGS V.4.2.0, through the “gemtc” package in R language (V.3.6.1) to conduct the NMA in a Bayesian hierarchical framework. Three chains with different initial values will be run simultaneously. For each analysis, the inference will be based on 150,000 iterations of Markov Chain Monte Carlo after a 50000 iteration burn-in period. Trace plots and Brooks–Gelman–Rubin diagnostic plots will be used to assess convergence.^[25]^ Deviance information criterion statistics and leverage plots will be used to assess the random effects model fit and ensure that the overall fit is adequate.^[26]^

A graphic representation of the network will be used to assess the strength of the evidence, which shows the number of articles from which the information presented comes (treatment nodes), the comparisons that have direct comparisons, those that present indirect or mixed comparisons and the number of patients with different comparisons, in such a way that confidence in the results will be increased.^[27]^

We will report our findings with probability statements of the intervention effects. Probability rankings allow us to report a chance percentage for which interventions rank higher.^[28]^ However, simplifying the results of a network down to probabilities can lead to misinterpretations, specifically when particular comparisons (i.e., nodes) are not well connected or when the quality of evidence varies between comparisons.^[29,30]^ Following the display of the rank probabilities using a rankogram, we will use the surface under the cumulative ranking (SUCRA) line to aid in the interpretation of the relative effect of the interventions. An intervention with an SUCRA value of 100 implies that treatment is certain to be the best, whereas an intervention with 0 is certain to be the least effective.^[28]^ Since most interventions listed above are combinations of other interventions, we also intend to run a component-level analysis, given that enough studies per component will be available.

### 2.11. Assessment of inconsistency

To measure the inconsistency between direct and indirect evidence, the node-splitting method will be used, which is a straightforward interpretation, contrasting estimates from both direct and indirect evidence.^[31]^ Values of *P* < .05 indicate an inconsistency between direct and indirect estimates in a specific closed loop.

### 2.12. Sensitivity analysis

We will assess the robustness of our results through a series of sensitivity analyses: the exclusion of trials with a high risk of bias and, the iterative removal of one study at a time.

### 2.13. Subgroup analysis and network meta-regression

From a clinical point of view, MG differs among patients in terms of the distribution of muscle weakness, presenting as either ocular or generalized MG,^[32]^ and in terms of severity, ranging from mild to severe or life-threatening.^[33]^ It also differs in thymus pathology, varying from normal to hyperplasia or thymoma.^[34]^ In response to immunosuppressive and immunomodulatory therapies, some patients become refractory to conventional drugs.^[3,35]^ Classifying patients according to their clinical and immunological characteristics allows a better understanding of the disease and helps to select the most appropriate treatment.^[36]^ If important heterogeneity and/or inconsistency is found, the possible factors will be explored. If sufficient studies are available, network meta-regression or subgroup analyses will be performed on patients in accordance with their clinical and immunological characteristics.

### 2.14. Assessment of publication bias and small-study effects

Publication bias will be examined using Begg’s and Egger’s funnel plots when applicable. In addition, the contour-enhanced funnel plot will be obtained as an aid to distinguish asymmetry due to publication bias. Small-study effects will be tested using a network meta-regression model that distinguishes studies based on their size.

### 2.15. Software

Pairwise meta-analyses will be conducted using STATA software (V.14.1 Stata/SE, Stata Corporation, TX). The Bayesian meta-analyses will be performed using JAGS V.4.2.0, through the “gemtc” package in R software (V.3.4.4). The “Network Graphs” package will also be used to produce some of the figures, such as the geometry (network) plots, rankograms, SUCRA plots, and comparison-adjusted funnel plots.

### 2.16. Assessment of the confidence in the evidence from NMA

Confidence in the relative treatment effect estimated in the NMA for the primary outcome will be evaluated using confidence in network meta-analysis (CINeMA), which is a web application (http://cinema.ispm.ch/model/CINeMA_paper.pdf).^[37]^ The CINeMA tool will be used to assess the overall quality of the body of evidence for the primary outcomes based on within-study bias, indirectness, imprecision, heterogeneity, incoherence, and reporting bias. The quality of evidence will be classified according to the Grading of Recommendations, Assessment, Development, and Evaluations (GRADE) group into 4 levels: high, moderate, low, and very low quality.^[38]^

## 3. Discussion

MG is clinically heterogeneous and exhibits a variable treatment response. Hence its treatment should be, as much as possible, personalized and possibly falling into precision medicine.^[39]^ All statements comparing the merits of one immunosuppressive drug with another must be tempered by the potential limitations of the methodology,^[40]^ the complexity of specific patient populations, and the uncertainties that might result from the choice of dose or treatment setting. Notwithstanding these limitations, the findings from this NMA will represent the most comprehensive currently available evidence to guide the initial choice of pharmacological treatment for MG. We hope that the results will assist in shared decision-making between patients and their clinicians.

## Acknowledgments

The authors express their gratitude to all advisors for this study.

## Author Contributions

Peng Xu and Ying Zhang contributed equally to this work.

**Conceptualization:** Peng Xu, Zhiguo Lv, Tianying Chang, Tianye Lan, Jian Wang.

**Data curation:** Li Jiang, Yibin Zhang, Dongmei Zhang, Hanying Xu, Ying Zhang, and Zhen Hua.

**Formal analysis:** Tianying Chang, Peng Xu, Li Jiang, Ying Zhang, Yingzi Cui.

**Investigation:** Dongmei Zhang, Yibin Zhang, Hanying Xu, Jing Lu, Ying Zhang.

**Resources:** Dongmei Zhang, Yibin Zhang, Hanying Xu, Jing Lu, Zhen Hua, and Jinhui Tian.

**Methodology:** Yingzi Cui, Jinhui Tian, Jihui Ma.

**Validation:** Li Jiang, Qingxia Huang, Chengfei Gao, Ying Zhang, Jinhui Tian.

**Visualization:** Li Jiang, Qingxia Huang, Chengfei Gao, Ying Zhang, Jinhui Tian..

**Software:** Peng Xu, Tianying Chang, Zhen Hua, Chengfei Gao, Yingzi Cui, Jinhui Tian.

**Supervision:** Jihui Ma, Jian Wang.

**Writing—original draft:** Peng Xu.

**Writing-review and editing:** Peng Xu, Ying Zhang, Jihui Ma, and Jian Wang.
